# Case report: Reconstruction of a complex maxillofacial gunshot defect using a titanium patient-specific implant in a dog

**DOI:** 10.3389/fvets.2022.1050568

**Published:** 2022-11-10

**Authors:** Myungryul Yang, Jinsu Kang, Namsoo Kim, Suyoung Heo

**Affiliations:** Department of Surgery, College of Veterinary Medicine, Jeonbuk National University, Iksan-si, South Korea

**Keywords:** gunshot wound, reconstruction, three-dimensional, patient specific implant, dog

## Abstract

This report describes the surgical reconstruction of large maxillofacial defect caused by a short-range gunshot injury in a dog using titanium patient-specific implant (PSI). A 3-year-old male Wolf Shepherd was admitted for a large right facial defect with right nasal cavity exposure caused by a gunshot injury. Radiographic examination revealed severe loss of the right maxillary, nasal, and incisive bones, multiple fractures of both left and right palatine bones, and a comminuted fracture of the right mandible. Initial surgical procedure included computed tomography (CT) imaging for three-dimensional (3D) implant design. Open wound management was maintained for 18 days until the fresh granulation tissue fully covered the wound bed. The implant was designed in a “hand grasping shape” to cover the defect, align multiple fractured palatine bones, and make a snap fit function. Multiple holes, including cortical screw holes, were added to the final design. The implant was printed on a titanium alloy. Surgical application of titanium PSI was performed 19 days after the primary surgery. A free sublingual mucosal graft was used to reconstruct the mucosal layer of the right nasal cavity. The mucosa was then covered with collagen membrane to strengthen the structure of the nasal cavity. Blunt dissection of the hard palate mucoperiosteum above the palatine process and palatine bones, soft tissue above the maxilla was performed, and the 3D printed titanium implant was fastened in a preplanned position. The facial soft tissue defect was reconstructed, and the titanium PSI was covered using an angularis oris cutaneous flap. Partial flap necrosis occurred in the rostral aspect, and the wound was managed to heal by a second intension. Flap dehiscence at the junction of the flap and hard palate mucoperiosteum occurred with exposure of the implant 2 days postoperatively. Multiple attempts to close the defect failed, and the owner wanted to stop treatment. Healthy granulated tissue was observed proximal to the implant. The defect no longer increased in size and did not show any noticeable complications related to the defect at 60 days after titanium PSI application, and the dog was discharged. Six months post-operatively, the dog remained active with great appetite, gained weight, and showed acceptable facial symmetry without enlargement of the implant exposure or any implant-related problems.

## Introduction

Gunshot wounds represent a small portion of traumatic injuries in veterinary medicine and are potentially devastating, especially shots to the head (1). Bone and soft tissue loss requires extensive surgical intervention and postoperative care (2). Reconstruction of maxillofacial defects is extremely challenging because of the complex anatomy, uniqueness of each defect, and chance of infection (3). The goal of craniofacial reconstruction includes anatomical, functional, and aesthetic restoration (4).

Autologous bone grafts for hard-tissue reconstruction remain the gold standard because of their bio-friendliness (5, 6). However, such surgical options have critical disadvantages, including donor-site morbidity, prolonged anesthesia time, availability in limited quantities, unpredictability of bone graft resorption, and the need for manual sculpting of the graft intraoperatively (7–9).

Application of recombinant human bone morphogenetic proteins (rhBMP-2) as a regenerative technique combined with mandibular reconstruction has been reported in several cases, but to our knowledge, the use of rhBMP-2 in maxilla reconstruction has not been reported (10).

Three-dimensional (3D) printing, a novel technique in human reconstructive medicine, has advanced over the past three decades and has been applied in the production of patient-specific implants (11, 12). Patient-specific implants have gained importance in treating maxillofacial defects owing to their precise adaptation, reduced surgical times, and better cosmesis (4, 11–14).

In the veterinary literature, there are multiple clinical reports of reconstructive techniques without use of PSI (mini plates, bone grafts) for maxillofacial defects (10, 11, 13, 15–18). Two case reports using 3D printing technique following tumor removal has been published (19, 20). One case used a titanium PSI for a mandibular segmental defect reconstruction, and the other used a 3D printed polycaprolactone (PCL) scaffold as a reconstruction method of the maxilla. In our case, a more rigid implant was needed because of the large bone defect with multiple fractures of the upper jaw, followed by a gunshot injury.

This report describes the workflow of a novel snap fit PSI design and the successful reconstruction of a large maxillofacial gunshot defect by applying a titanium PSI in a dog.

## Case report

A 3-year-old intact male Wolf Shepherd dog weighing 40 kg presented with a severe right maxillofacial defect caused by a gunshot wound on the previous day. On presentation, a large portion of the tissue in the right muzzle area was severely damaged and lost. The nasal and oral cavities were observed through the defect. During breathing, the air flow was disturbed by the open defect.

**Figure 1:**
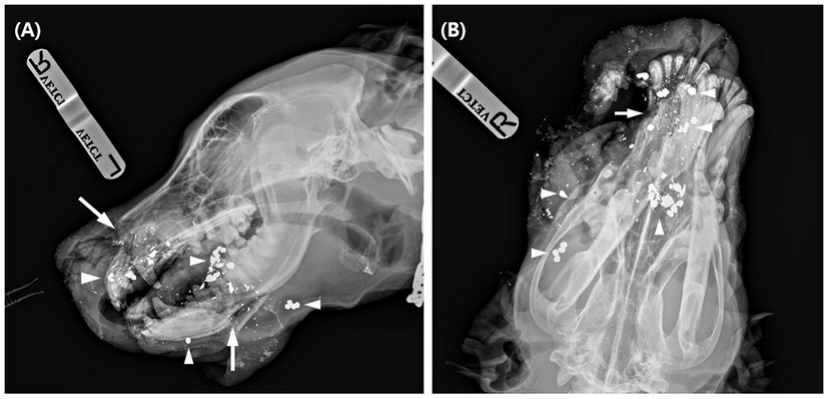
Lateral **(A)** and dorsoventral **(B)** radiographs of the dog at presentation. On the lateral view, there are some radiolucent fracture lines (arrow) at the incisive bone, mandible, and multiple metal opacities (arrowhead) around the facial area. In the dorsoventral view, bone and soft tissue loss in the right rostral area was evident.

Preoperatively, blood analysis, including serum biochemistry, complete blood count, and blood gas analysis, was performed. There was a mild elevation of the white blood cell (WBC) count at 25.3 × 10^9^/L (reference range, 6–19 × 10^9^/L) with other values within the normal range. Radiographic skull examination (HF-525 PLUS; Ecoray, Seoul, Korea) revealed partial loss of the right incisive, cranial part of the right maxilla, and nasal bones. Multiple fractures of both left and right palatine bones and a butterfly fragment fracture of the right mandible were noticeable. More than twenty-five 3 x 3 mm round metal opaque materials suspected to be scattered bullet fragments were also found over the entire facial area (Figure 1).

Owing to the severity of the gunshot wound and multiple fractures with a large defect of the right maxillofacial bones, the owner was informed of the risks and benefits of the therapeutic options available (3D reconstruction and maxillectomy). Considering the dog's quality of life and the potential inability to eat by himself if maxillectomy has been selected, the owner chose to proceed with the reconstruction of the defect.

**Figure 2:**
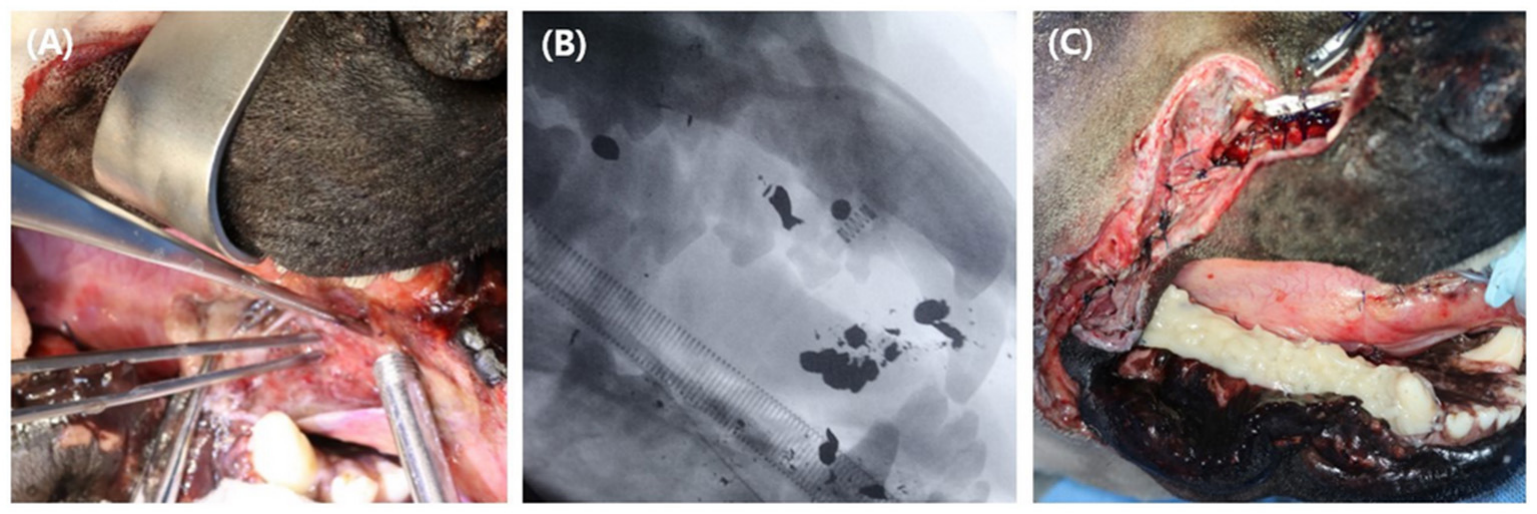
Emergency surgery was performed to debride devitalized tissues, to extract as many bullet fragments as possible and to stabilize fractured bones temporarily. Extraction of the bullet fragments **(A)** were made under fluoroscopic guidance **(B)**. A continuous stout loop interdental wire was applied at the right mandibular teeth and the fixation was reinforced with temporary resin over the area **(C)**.

Emergency surgery was performed under general anesthesia to remove scattered bullet fragments, debride non-vital tissues, and temporarily fix the fractured mandible. Butorphanol (Butophan, Myungmoon Pharm Co., Seoul, Korea) at 0.2 mg/kg IV (intravenous), midazolam (Midazolam, Bukwang Pharm Co., Seoul, Korea) at 0.2 mg/kg IV were administrated as preanesthetic medication. General anesthesia was induced with 6 mg/kg IV propofol (Provive 1%, Myungmoon Pharm Co., Seoul, Korea) and maintained with sevoflurane (Sevofran, Hana Pharm Co, Seoul, Korea). Cefazolin (Cefazolin sodium, Korus Pharm Co., Chuncheon, Korea) at 25 mg/kg IV was administered every 90 min perioperatively. For local anesthesia, caudal maxillary (infraorbital foramen) and mandibular (mandibular foramen) nerve block was made by injecting 0.5% bupivacaine at dosage volume of 1cc for each foramen. Bullet fragments were removed as much as possible under fluoroscopic guidance for 30 min (Figures 2A,B). Three large bullet fragments remained after surgery, but two were removed during open wound management. Soft tissue with suspected viability around the maxilla was aggressively debrided and flushed using sterile saline. The rostral part of the upper muzzle was left undisturbed as far as possible for future soft tissue reconstruction. The remaining soft tissues were sutured at the anatomical position using interrupted horizontal mattress pattern augmented by rubber stents as a tension-relieving technique. Incisive fractures and right mandibular fractures were fixed with continuous stout loop interdental wiring, reinforced with intraoral temporary resin (Luxatemp Automix Plus, DMG, New Jersey, USA) from the mandibular second molar teeth to the canine teeth (Figure 2C). A 24-Fr thoracic catheter (PVC thoracic catheter, Sewon medical, Chungnam, Korea) was used as an esophageal tube, exiting the left mid-cervical region percutaneously. CT imaging (Alexion, TSX-034A, Canon Medical Systems Europe B.V., Zoetermeer, Netherlands) of the facial area was performed immediately after surgery for 3D implant design.

Butorphanol–lidocaine–ketamine (butorphanol at a dose rate of 0.02 mg/kg/h, lidocaine at a dose rate of 1.5 mg/kg/h, ketamine at a dose rate of 0.6 mg/kg/h) were administered at a constant rate for post operative analgesia. While recovering from surgery, the dog seemed anxious and screamed with aggressive movements. A high respiratory rate of 120–180 and respiratory distress occurred from the opening of the oral and nasal cavities; heart rate increased from 100 to 220 bpm, and blood pressure was mildly elevated (systolic 110–130 mmHg).

Medetomidine (Tomidin, JSK, Goyang, Korea) was administered at a dose of 10 μg/kg as rescue analgesic and sedative. While the dog was sedated, additional nerve block was made as described above. Multiple sedation treatments were administered as needed until the vital signs stabilized three days postoperatively.

**Figure 3:**
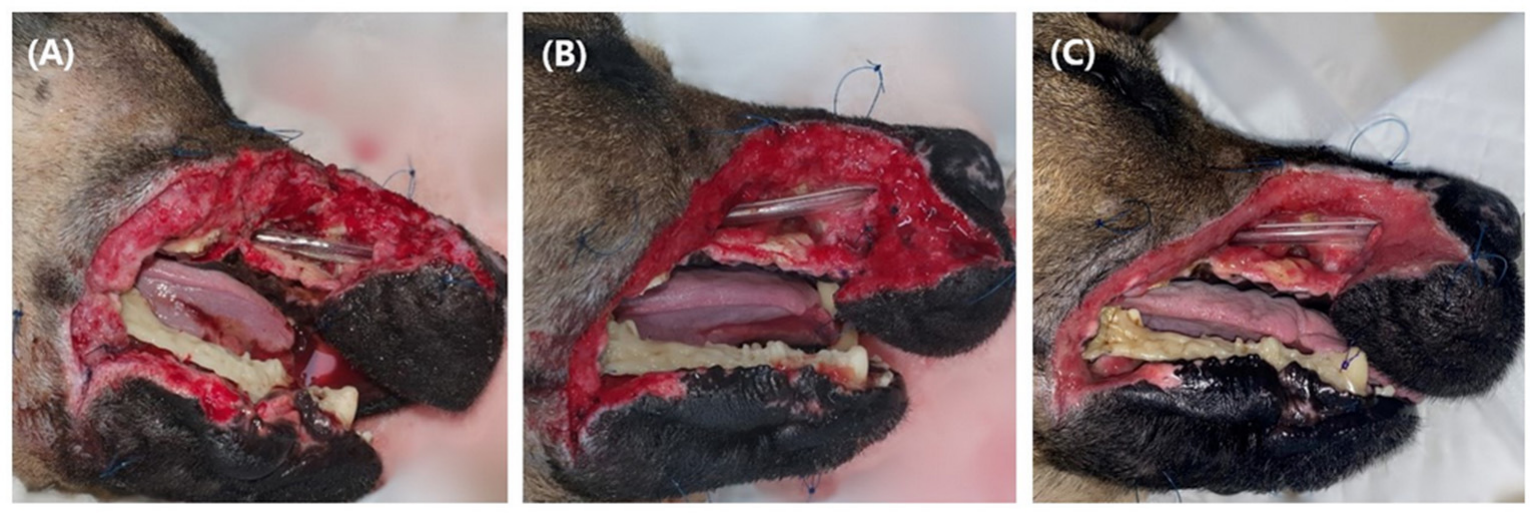
Until healthy granulation tissue filled the defect, the wound was managed wet-dry for 15 days. Two days after open management **(A)**, 5 days after open management **(B)**, 15 days after open management **(C)**. Fresh granulation tissue started to form at some parts of the defect at 5 days, and at 15 days after open management, the wound was fully covered with granulation tissue. Note the endotracheal tube placed through the open nasal cavity (arrow), and loose sutures (arrowhead) around the defect for tie over bandage.

Four days after the first surgery, a second surgery was performed for additional debridement of the upper lip and incisive bone, and an endotracheal tube (I.D. 4.5 mm) was installed through the nasal defect into the nasopharynx and sutured at the nostril with 3-0 nylon to secure the nasal airflow and improve breathing. The dog recovered from anesthesia without any complications. Until healthy granulation tissue filled the entire wound bed, wet dry bandaging was performed twice daily under mild sedation with medetomidine 10 μg/kg IV for 15 days (Figure 3).

**Figure 4:**
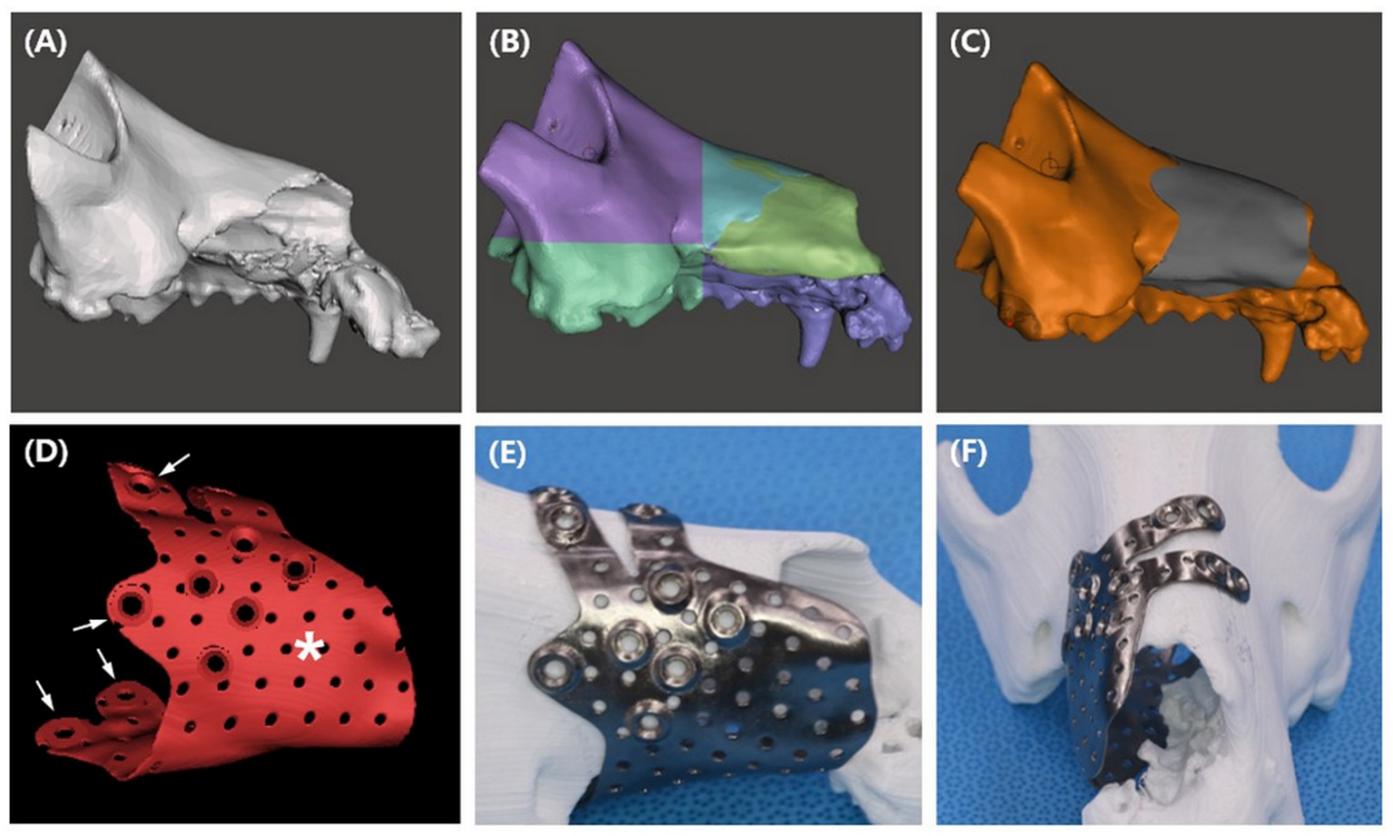
PSI design procedures **(A–D)**, and presurgical application of the titanium PSI on the printed PLA bone. STL file of the maxilla **(A)** was sectioned to smaller parts **(B)**, mirroring of the left maxilla reconstructed the right maxilla (yellow green area). Implant shape was offset from the bone **(C)**, multiple pores and 2.4 cortical screw holes were added to the design **(D)**. Finger parts of the design (arrow) allows the PSI to snap fit to the bone, the palm part (asterisk) covers the bone defect. After receiving the titanium implant, simulation of the implant was performed on a PLA printed bone model **(E,F)**.

The implant was designed based on CT scans. First, CT images were saved in Digital Imaging and Communications in Medicine (DICOM) format. Subsequently, using 3D medical image processing software (Mimics Innovation Suite 23.0, Materialize, Leuven, Belgium), the DICOM file was converted into a 3D format, Standard Tessellation Language (STL). The STL file was then transported to 3D design software (Mesh mixer, Autodesk, California, USA) to build a patient-specific implant (Figure 4A).

The implant design was aimed at covering the maxillofacial defect and maximizing contact with the skull for rigidity. Additionally, a snap fit design that allowed the implant to easily snap into the bone defect was considered to eliminate the need for further surgical complexity. To construct a snap fit, the implant was designed to look like a c shape on a transverse plane and the finger part was designed to hang over the left nasal bone (Figure 4F).

Due to the large defect of the right maxilla, the anatomical shape of the implant was designed from the intact left maxilla. By creating a mirror image of the left maxilla in the median plane, the right maxilla was reconstructed (Figure 4B), and the implant shape was offset from the reconstructed area (Figure 4C). The implant was designed in a “hand grasping” shape, composed of the palm and fingers. A large defect was covered by the palm part of the implant, and a 180-degree rigid bond with the skull and a snap fit function to the skull were obtained by the finger part. To ensure strength and biocompatibility, an implant thickness of 0.8 mm was selected, and multiple pores with a diameter of 1 mm were randomly created. Finally, 2.4/2.7 cortical screw holes were added at the tip of the finger and palm parts (Figure 4D).

The implant design was sent to a titanium printing manufacturer (Anibone, Cusmedi, Suwon, Korea) and printed using titanium alloy. After receiving the titanium PSI, simulation surgery was performed to confirm the accuracy of the implant. The bone model was printed in polylactic acid (PLA) filament using a fused deposition modeling (FDM) printer (MakerBot Plus, MakerBot Inc., NY, USA) at our medical center (Figures 4E,F).

**Figure 5:**
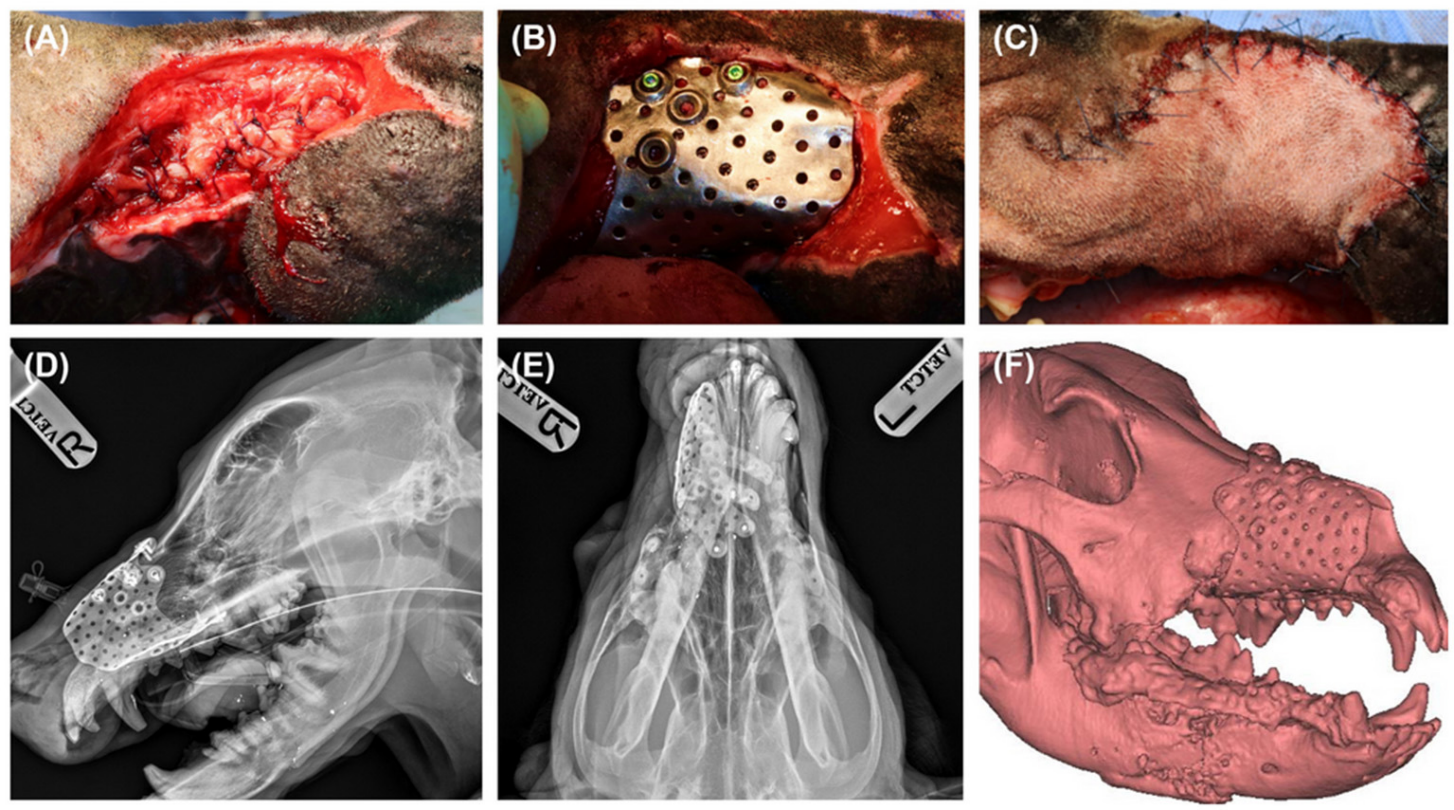
Sublingual mucosa graft was applied and undermined soft tissue around the defect was sutured over the graft to secure reconstructed nasal cavity **(A)**. Titanium PSI was seated percutaneously, and screws were placed under fluoroscopic guidance **(B)**. PSI was fully covered with angularis oris flap **(C)**. Post operative radiographs, lateral **(D)** and dorsoventral **(E)** view, and 3D reconstruction of post operative CT scan revealed precise fit of the implant **(F)**.

Fifteen days after the second surgery, maxillofacial reconstruction was performed by installing the titanium PSI. Before installation of the PSI, the free sublingual mucosal graft was grafted for nasal mucosal reconstruction. The mucosa was then covered with a type 1 collagen membrane (Lyoplant, B.Braun, Melsungen, Germany) to strengthen the structure of the nasal cavity. The soft tissue around the maxillary defect was undermined and sutured over the grafted sublingual mucosa (Figure 5A). The implant was then seated percutaneously and a perfect snap fit was obtained by pushing the PSI through the planned area (Figure 5B). Screws were placed under fluoroscopic guidance using a minimally invasive technique. A stab incision was made over the screw holes, and 2.4 cortical screws (2.4/2.7 ALPS, Able vet, Jeonju, Korea) were applied to the titanium PSI. To cover the implant and reconstruct the facial soft tissue, the angularis oris cutaneous flap was sufficient for full coverage of the rostral extent of the defect (Figure 5C). Postoperative radiographs and CT tomography revealed a precise fit of the PSI (Figures 5D–F).

Three days after titanium PSI application, partial necrosis occurred in the rostral part of the flap. The defect area was 1 x 2 cm in size, and the wound was managed open for secondary wound closure and recovered without additional complications. Flap dehiscence occurred 2 days postoperatively between the hard palate mucoperiosteum and angularis oris cutaneous flap, at the level of the maxillary first premolar teeth to the third premolar teeth, which resulted in implant exposure. Because the dehiscence was located at the rostral part of the head, the adjacent skin used to cover up the defect was limited. Surgical revision to close the dehiscence was performed using a combination of a palate mucoperiosteum pedicle flap and buccal mucosa free graft. Graft failure and dehiscence reoccurred 6 days after the revision. Another attempt was made with a tubular subdermal flap from the dorsal cervical area to close the defect; however, tube necrosis occurred seven days after subdermal tube creation. The remaining part of the tube was sutured to the right buccal area for spare. No further attempts were made after tubular subdermal flap failure because the owner declined additional surgical revision of the defect. The flap defect did not increase in size. The soft tissue under the implant was intact and granulation tissue filling the implant hole was observed. Signs of complications related to implants, such as infection or oronasal fistula formation, did not occur because of dehiscence. The esophagostomy tube was removed, and the dog was fed orally 50 days after titanium PSI application (70 days after the first surgery). No complications occurred during oral administration of liquid and soft diet for 7 days, and the dog was discharged.

**Figure 6:**
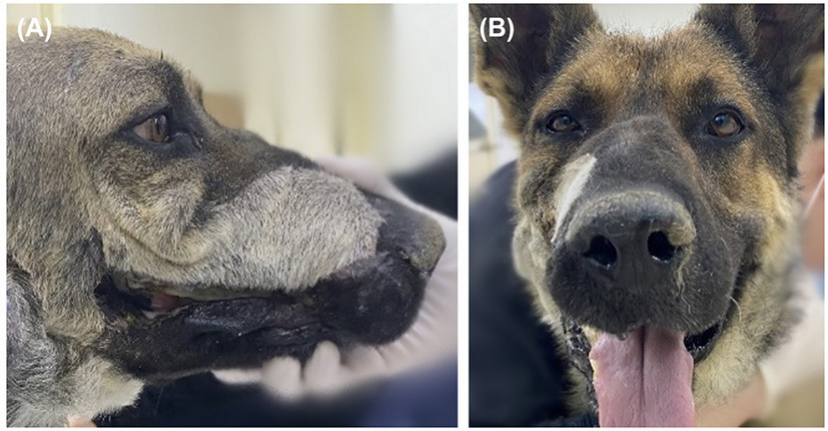
Evaluation of the cosmesis of the reconstruction 6 months after PSI application. Except for the hair color and direction change resulted from angularis oris cutaneous flap seen on lateral view **(A)**, facial symmetry was perfect on front view **(B)**.

**Figure 7:**
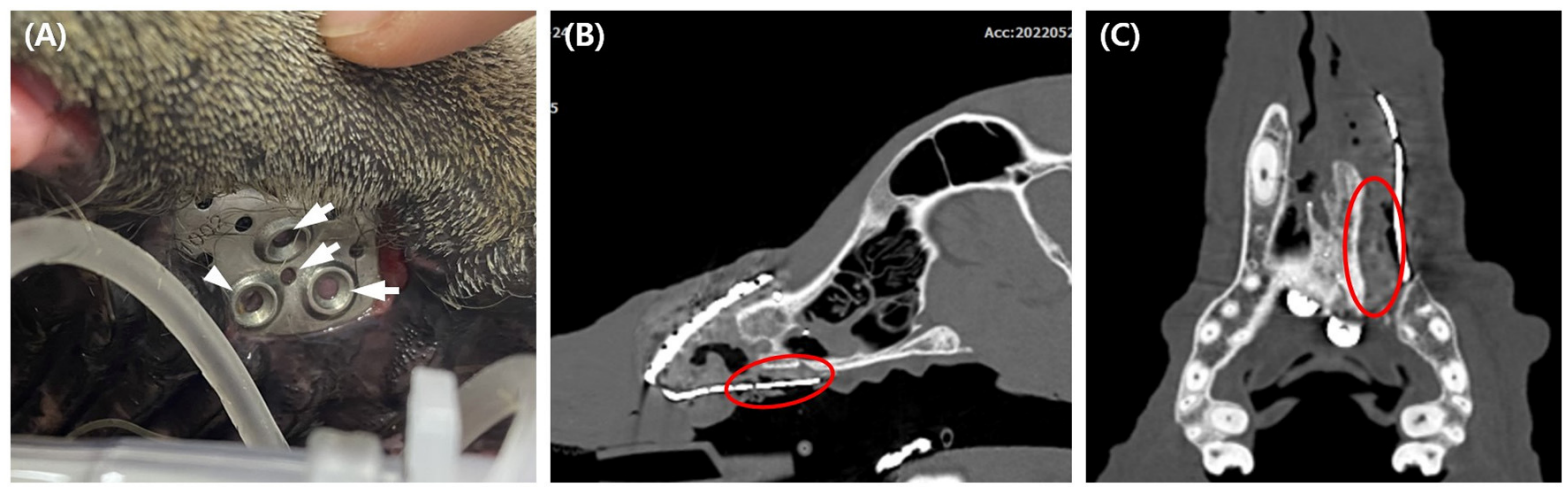
Six months postoperative evaluation of the implant exposure. Note the pink soft tissue under the implant hole (arrow), and the pigmented tissue suggesting palatal epithelium under one hole (arrowhead) **(A)**. CT taken 6 months after surgery revealed soft tissue formation presumed to be palate mucoperiosteum (red circle) **(B,C)**.

Oral examinations were performed on a monthly basis to evaluate facial symmetry, facial nerve function, implant exposure size, nasal discharge or obstruction, possible flap-related problems, and implant-related problems. Facial symmetry was achieved 3 months after the flap surgery and was maintained through the last evaluation at the 6 month follow-up (Figure 6). Because the dog had a habit of biting shoes or a feeding bowl, minor enlargement of the implant exposure was found (Figure 7A). The dog did not show any other flap- or implant-related complications. Six months after titanium PSI application, CT imaging revealed healing of the fractured mandible, and the temporary resin and interdental wire were detached. Also, under anesthesia, nasal endoscopy (Lscope, Seplou, Loganville, USA) confirmed full recovery of the nasal mucosa (Supplementary Video 1). The dog remained vigorous with a great appetite, gained weight, and showed acceptable facial symmetry without enlargement of the implant exposure.

## Discussion

Reconstruction of maxillofacial defects is extremely challenging because of the complex anatomy, unique nature of each defect, and chance of infection (3). Surgical options such as autologous bone grafting or internal fixation with the application of regenerative materials are also available (5, 6). Autologous bone grafting remains the gold standard in human reconstructive medicine but has critical disadvantages, including donor-site morbidity, prolonged anesthesia time, limited availability, unpredictable bone graft resorption, and the need for manual sculpting of the graft intraoperatively (7–9). With the emergence and advancement of 3D printing techniques, patient-specific implants have gained importance in these maxillofacial defects due to their precise adaptation, reduced surgical times, and better cosmesis in human surgery (4, 12, 13, 17). 3D scaffold with polycaprolactone (PCL), a biodegradable polymer, and titanium implants are commonly used for facial implants (19).

In veterinary practice, a study of maxillary bone reconstruction with a 3D PCL scaffold following tumor removal has been described (21). According to the study, bone defects were due to removal of a mass with a relatively small size (2.0 x 3.1 cm). The palatine bone was intact and the defect was surrounded by the maxilla, which made it possible for the scaffold to fit into the defect. In this case, a high-velocity gunshot injury resulted in destruction of the incisive bone, palatine bone, right maxilla, and mandible. The implant needed to hold the palatine fragments together, cover a large maxillofacial defect, and secure the reconstructed nasal passage. Titanium was selected as the implant material because of its biocompatibility, strength, and osseointegrative properties (4).

The goal of craniofacial reconstruction includes anatomical, functional, and aesthetic restoration (22). Our titanium PSI was designed to reconstruct the maxillary defect, stabilize multiple fractures of palatine process and both sides of palatine bones, and seal the nasal cavity. To achieve this goal, the implant was designed with a unique hand-grasping shape. The implant consists of two parts: the palm and fingers. The palm part reconstructed the maxillary defect, secured nasal passage, and held the palatine fragments together. The finger part was made to grab the intact bone parts rigidly with minimal interference from the blood supply and to snap fit and hold on to the bone.

One of the advantages of the medical application of 3D technology is the use of 3D models prior to surgery, which improves planning and shortens the duration of the surgery (23). In our case, the skull of the patient was printed using PLA for simulated surgery and intraoperative guidance. We also confirmed that the titanium PSI perfectly fits onto the bone surface and selected the best direction to install the implant. Simulation of the procedure and snap fit design significantly shortened the time required for titanium PSI application. The time taken for titanium PSI installation was <30 min.

Soft tissue reconstruction for large nasal and facial wounds is limited because scarcity of regional direct cutaneous pedicles able to support an enough sized skin flap for primary closure (24). In this case, we planned to cover the titanium PSI with a cutaneous angularis oris axial pattern flap because it has an independent blood supply (23). Superficial temporal, angularis oris, caudal auricular, and superficial cervical axial pattern flaps are described as facial reconstructive axial pattern flap options. The angularis oris flap was applied because the defect included the rostral end of the face in this patient (23, 24).

Complications of maxillofacial fracture reconstruction include nasal passage- and implant-related problems (22). The nasal passage was inspected for possible obstruction and nasal discharge. At the latest recheck 6 months postoperatively, there were no signs of nasal discharge or discomfort when breathing through the nose when each nostril was blocked one at a time. Two months postoperatively, rhinoscopy revealed regrowth of nasal mucosa without obvious oronasal or nasocutaneous defects. Oral examination and CT tomography were performed for implant-related complications. Implant exposure in the oral cavity occurred 3 days after titanium PSI installation. Attempts for direct closure, palatal rotational flaps, and tubular subdermal flaps have failed.

Exposure of titanium PSI to the oral cavity was the main postsurgical concern in our study. Bacterial contamination of implants is a major cause of implant failure (21). Implant exposure is treated by removing the implant to prevent long-term negative effects and resolve complications (10, 25). In this case, the implant was not removed, and other than plate exposure there were no complications from the implant noted 6 months post-operatively. Although the postoperative evaluation period was short, the implants were not removed because of (1) the rich vascularization of the oral anatomy and (2) the thin, porous design of the implant provides host-cell integration that reduces bacterial contamination and infection (21). Additionally, a postoperative CT scan at 6 months revealed soft tissue presumed to be palate mucoperiosteum growth between the exposed implant and palatine bone, which could have worked as a physical barrier (Figures 7B,C).

Gunshot injuries to the head often result in substantial fractures and possible significant tissue loss and require multi stage procedures (22, 26). Because of the time-consuming nature and large expense of treatment and the potential decrease in quality of life after treatment, there are limited research on gunshot-acquired facial defect reconstruction in veterinary surgery (2, 20). Maxillectomy could be a treatment option; however, postoperative complications such as abnormal salivation, cheilitis, dermatitis, and cosmetic defects are common because of the nature of radical excision (27, 28). In addition, willingness to undertake decent aftercare is a crucial point for successful treatment after maxillectomy (27). In our case, by reconstructing the rostral structure of the maxilla with titanium PSI, none of these complications were present, and additional aftercare was not necessary since the dog recovered to chew and eat by himself.

This report describes a 3D design procedure for a snap fit titanium PSI and surgical reconstruction of a dog with a large maxillofacial defect caused by a short-range gunshot injury by application of a titanium PSI. Surgical reconstruction results in satisfactory functional and aesthetic outcomes. Surgical repair using titanium PSI could be considered an option for complex maxillofacial gunshot defects.

## Data availability statement

The original contributions presented in the study are included in the article/Supplementary material, further inquiries can be directed to the corresponding author.

## Author contributions

MY and SH: conception, design, and draft of the manuscript. MY, JK, and SH: main clinician during case presentation and performed surgery. MY, NK, and SH revised the article for intellectual content and final approval of the completed article. All authors have contributed to the manuscript and approved the submitted version.

## Funding

This research was financially supported by the Ministry of Small and Medium-sized Enterprises (SMEs) and Startups (MSS), Korea, under the Regional Specialized Industry Development Plus Program (R and D, S3244754) supervised by the Korea Technology and Information Promotion Agency (TIPA).

## Conflict of interest

The authors declare that the research was conducted in the absence of any commercial or financial relationships that could be construed as a potential conflict of interest.

## Publisher's note

All claims expressed in this article are solely those of the authors and do not necessarily represent those of their affiliated organizations, or those of the publisher, the editors and the reviewers. Any product that may be evaluated in this article, or claim that may be made by its manufacturer, is not guaranteed or endorsed by the publisher.

## Supplementary material

Supplementary Video 1 Under anesthesia, right nasal endoscopy was performed full recovery of the reconstructed nasal wall and mucosa was confirmed (left side of the video).
